# Understanding the Impact of Population and Cancer Type on Tumor Mutation Burden Scores: A Comprehensive Whole-Exome Study in Cancer Patients From India

**DOI:** 10.1200/GO.23.00047

**Published:** 2023-11-02

**Authors:** Nishtha AjitSingh Tanwar, Richa Malhotra, Aiswarya Padmaja Satheesh, Satya Prakash Khuntia, Peddagangannagari Sreekanthreddy, Linu Varghese, Srivalli Kolla, Pratik Chandrani, Anuradha Choughule, Priyanka Pange, Vinod Gupta, Vanita Noronha, Vijay Maruti Patil, Raja Pramanik, Sunila Kumar, Sandeep Peraje Nayak, Suresh Babu, Rohan Shetty, Madan Kantharaju, Pramod Shekarappa Chinder, Aruna Korlimarla, BS Srinath, Kumar Prabhash, Kshitij Datta Rishi, Hitesh Madan Goswami, Vidya Harini Veldore

**Affiliations:** ^1^ 4basecare Onco Solutions Pvt, Ltd, Bangalore, India; ^2^ Tata Memorial Centre, Mumbai, India; ^3^ Medical Oncology Molecular Laboratory, Tata Memorial Centre, Mumbai, India; ^4^ Department of Medical Oncology, Tata Memorial Centre, Mumbai, India; ^5^ Fortis Cancer Research Centre, Bangalore, India; ^6^ Yenepoya Medical College, Mangalore, India; ^7^ AARO Clinic, Bangalore, India; ^8^ Sri Shankara Cancer Hospital & Research Centre, Bangalore, India

## Abstract

**PURPOSE:**

The purpose of this study was to understand the impact of population diversity and geographic variation on tumor mutation burden (TMB) scores across cancers and its implication on stratification of patients for immune checkpoint inhibitor (ICI) therapy.

**MATERIALS AND METHODS:**

This retrospective study used whole-exome sequencing (WES) to profile 1,233 Indian patients with cancer across 30 different cancer types and to estimate their TMB scores. A WES-based pipeline was adopted, along with an indigenously developed strategy for arriving at true somatic mutations. A robust unsupervised machine learning approach was used to understand the distribution of TMB scores across different populations and within the population.

**RESULTS:**

The results of the study showed a biphasic distribution of TMB scores in most cancers, with different threshold scores across cancer types. Patients with cancer in India had higher TMB scores compared with the Caucasian patients. We also observed that the TMB score value at 90th percentile (predicting high efficacy to ICI) was high in four different cancer types (sarcoma, ovary, head and neck, and breast) in the Indian cohort as compared with The Cancer Genome Atlas or public cohort. However, in lung and colorectal cancers, the TMB score distribution was similar between the two population cohorts.

**CONCLUSION:**

The findings of this study indicate that it is crucial to benchmark both cancer-specific and population-specific TMB distributions to establish a TMB threshold for each cancer in various populations. Additional prospective studies on much larger population across different cancers are warranted to validate this observation to become the standard of care.

## Teaser

The study depicts significance of TMB as a biomarker, variable across cancers and populations/ethnicities.

## INTRODUCTION

**FIG A1:**
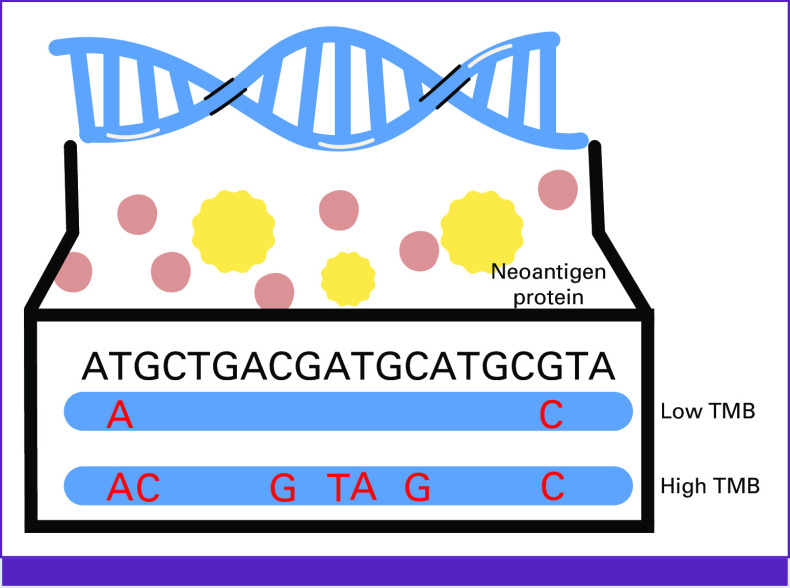
Schematic representation of TMB: The total number of somatic or acquired mutations per coding area of a tumor genome (mut/Mb) is known as TMB. TMB-High indicates an increased number of somatic mutations and therefore predicts a good response to immune checkpoint inhibitors. TMB, tumor mutation burden.

Improved treatment outcomes, alongside rapid advancements in immune checkpoint inhibitor (ICI) have revolutionized the clinical outcome in terms of quality of life, progression-free survival, and overall survival across multiple cancer types.^1^ As ICI becomes a more viable treatment option for patients with cancer, it is crucial to have a clear understanding of the biomarkers used to predict the response to ICI therapy. There are three major predictive biomarkers associated with ICI namely PD-L1 expression, microsatellite stability (MSI), and tumor mutation burden (TMB).^2,3^ While PD-L1 is a protein expression biomarker, MSI and TMB are genome-wide signatures derived from tumor DNA profiling. The overall somatic mutation load, accounted for within the coding regions of the tumor genome per megabase, is called TMB (Appendix Fig A1).^3-6^ The range of TMB scores, however, greatly varies across different cancer types, sequenced region size, nature of mutations (synonymous or nonsynonymous), choice of genes, variant filtration strategy to eliminate common polymorphisms in the population, and inherent biology of the tumor.^7^

**CONTEXT**

**Key Objective**
How does population diversity and geographic variation affect tumor mutation burden (TMB) scores across cancers and how can this influence the selection of patients for immune checkpoint inhibitor (ICI) therapy?
**Knowledge Generated**
In this milestone scientific study, we conducted whole-exome sequencing (WES) on 1,233 Indian patients with cancer across 30 cancer types, revealing a biphasic TMB score distribution as compared with Caucasian populations. The median TMB score varied significantly in four different cancers on comparison with TMB data generated from Western counterparts (TCGA), particularly in sarcoma, ovarian, head and neck, and breast cancers. Moreover, we established a reliable machine learning-based workflow for estimating TMB scores solely from tumor samples processed on whole exome.
**Relevance**
Our study emphasizes the need for benchmarking both population-specific and cancer-specific TMB thresholds to effectively stratify patients for ICI therapy response. Additionally, we demonstrated the practicality and precision of tumor-only WES in calculating TMB, reducing sequencing costs and eliminating the complexity associated with obtaining matched normal samples.

As per the global cancer statistics derived from Globocan, 2021, Asia contributes to more than 40% of the world's cancer burden, and India contributes more than 18% of global cancer incidence.^8^ As 85% of all cancers are somatic in origin, along with geographic variation, there are other factors, such as lifestyle (smoking, alcohol, predisposition to other diseases), occupational hazards, food, and ancestry, that contribute to increased risk of cancer incidence.^9^

Although, Asia contributes to 40% of the world's cancer burden, in oncology research, the representation of Asian patients in global clinical studies is <15%.^10^ Most of the US Food and Drug Administration (FDA) approvals for companion diagnostics are based on the research and findings of NGS panels derived from the non-Hispanic White Caucasian population. The major factor which contributes to this disparity includes the role of population-specific polymorphisms, tumor-specific mutations, and their aggressive behavior which varies across individuals in specific populations.

One of the recent studies led by Nassar et al clearly states that TMB calculated using standard methods developed and approved by the FDA is overestimated in the African population as compared with the East European population on the basis of real-world evidence from ICI outcome. According to the authors, nearly 21% of patients of European ancestry had false high-TMB misclassification; then again, almost 37% of Asian and 44% of African descent patients had misclassification of their TMB score.^10^ From this study, it is evident that ethnicity and geography play a significant role in the clinical outcomes. Therefore, it is warranted to establish these biomarkers in different populations to stratify patients who are eligible for ICI, thus avoiding treatment-related toxicities, and to reduce the cost of treatment. This study discusses the importance of understanding tumor genomic signature variation, TMB, in Indian versus western counterparts, to stratify patients who would respond to therapy with minimum toxicity for ICI.

In the past 2-3 years, there has been increasing evidence on the potential role of population and ethnicity affecting ICI outcomes in multiple cancers. One such clinical study on 207 patients (non–small-cell lung cancer [NSCLC] + head and neck squamous cell carcinomas) to evaluate the response to ICI with/without chemotherapy combination observed that racial or ethnic disparity had a significant impact on the objective response rate (ORR) as well as OR in these patients with cancer. The ORR for Hispanic (H) and Black (B) patients was lower compared with non-Hispanic White (W) patients although not statistically significant (H: 27.0%, B: 32.5%, W: 38.7%; H *v* W: *P* = .209; B *v* W: *P* = .398). When considering only patients treated with ICI monotherapy, the ORR for Hispanic patients dropped further to 20.7% while the ORR of Black and non-Hispanic White patients remained about the same (B: 29.3% and W: 35.9%, H *v* W *P* = .133; B *v* W *P* = .419). Immune-related adverse events were the lowest in the Hispanic population occurring in only 30% of patients compared with 40% of patients in the Black cohort and 50% of the non-Hispanic White cohorts.^11^

In this study, we used a whole-exome sequencing (WES)–based pipeline for TMB calculation in 1,233 Indian patients with cancer, with an indigenously developed strategy for the prediction of true somatic mutations, and adopted a robust nonguided machine learning approach, to understand the distribution of TMB scores. To the best of our knowledge, this is the largest clinical study of its kind from any one of the South Asian countries along with India. Predicting the TMB score distribution across different cancers has significant relevance not only in choosing the patient who has high chances of responding to ICI but also reduces the burden of treatment cost and toxicity for those patients who may not respond to ICI. We also performed a comparative analysis with publicly available The Cancer Genome Atlas (TCGA) data that constitute primarily Caucasians/East European ancestry to understand the role of genetic diversity and ethnicity.^12^

## MATERIALS AND METHODS

### Indian Patient Cohort

**FIG 1:**
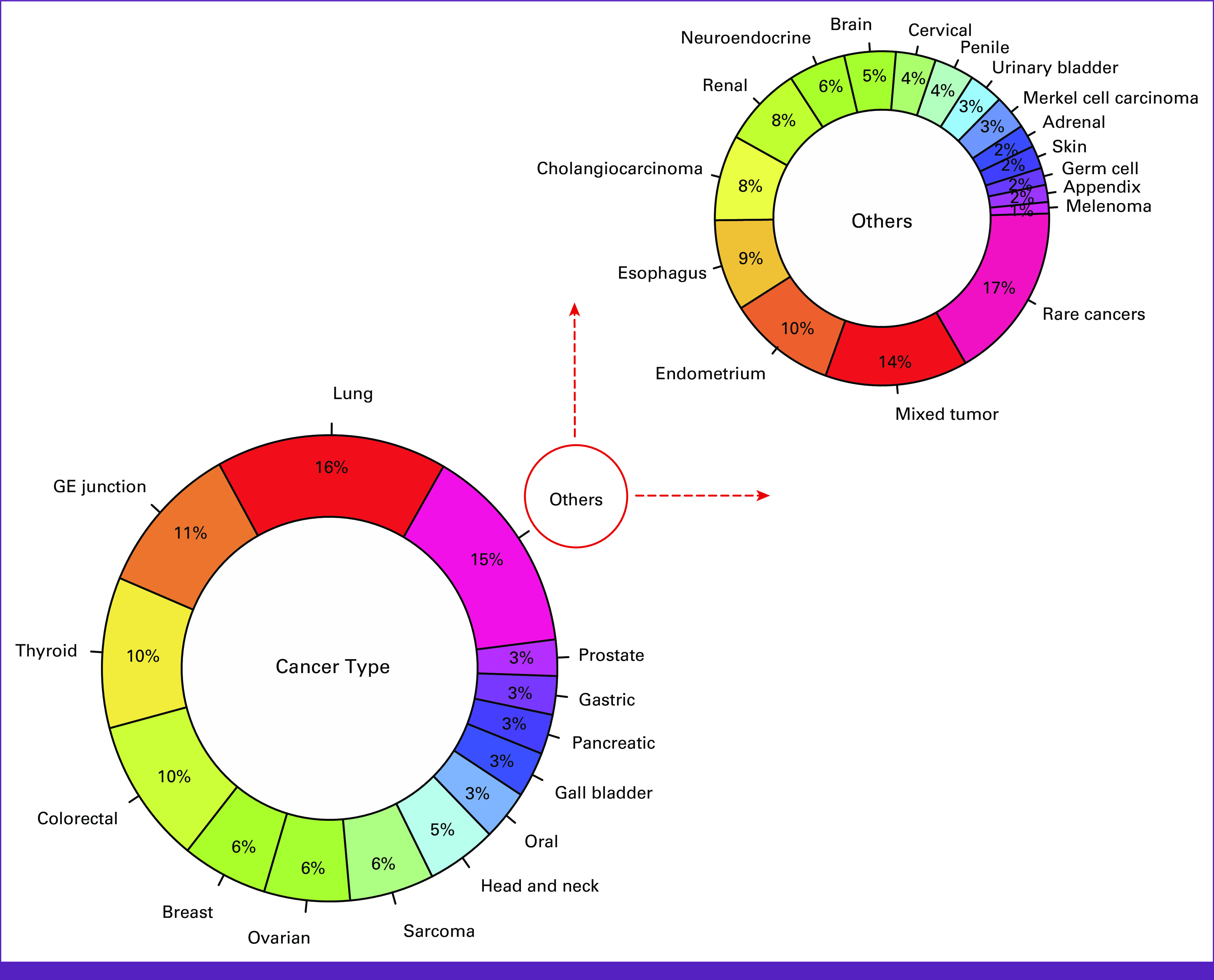
Cancer-wise distribution of the Indian cohort (N = 1,233) across 30 different cancer types sequenced and analyzed in-house for TMB estimation using whole-exome sequencing data. GE, gastroesophageal; TMB, tumor mutation burden.

The Indian cohort constitutes a total of 1,233 patient's tumors (from the advanced stage: stage III/IV) across 30 different cancer types, which were processed during December 2020 to January 2022 (Fig 1). This study was conducted according to the principles of the Declaration of Helsinki and as per the International Council on Harmonisation and Good Clinical Practice guidelines.^13,14^ All the data analyzed as part of this study are a retrospective analysis of patients with cancer, and written informed consent was obtained from these patients to use this deidentified information for research purposes. This study was approved by an independent ethics committee and review board (JCDC, India).

### Library Preparation and Sequencing

The formalin-fixed paraffin-embedded (FFPE) blocks with minimum tumor surface area ≥5 mm^2^ and tumor content ≥10% (ie, approximately, 150 viable tumor cells per high power field (HPF) on microscopy as per histological examination) were processed for genomic DNA extraction using All Prep FFPE DNA/RNA kit Cat. No. 80234 (Qiagen, Valencia, CA). Quality control (QC)–qualified DNA samples were processed for library preparation, which includes fragmentation, adapter addition, amplification, and capturing of exonic regions through overnight hybridization of exon-specific probes using Agilent DNA Prep with Enrichment kit (Cat. No. 5191-6874). The prepared libraries underwent QC analysis for the detection of library fragment size and concentration. The qualified NGS libraries were subjected to paired end (2 × 150 read length configuration) sequencing on the NextSeq Systems (Illumina Inc, San Diago, CA) at a mean coverage depth of 200× (Table 1).

**TABLE 1:**
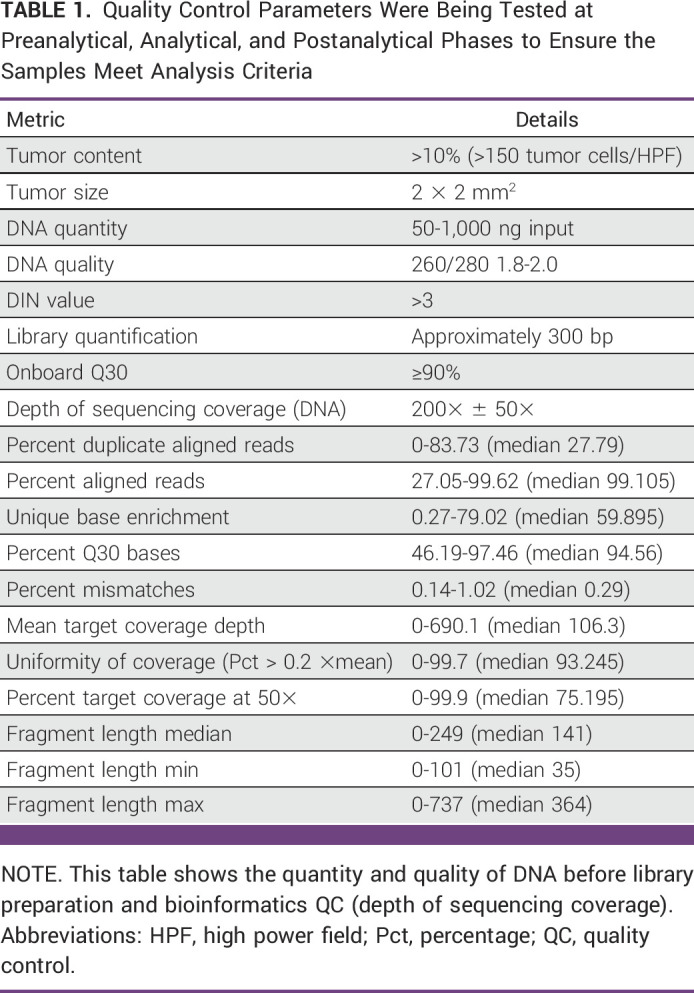
Quality Control Parameters Were Being Tested at Preanalytical, Analytical, and Postanalytical Phases to Ensure the Samples Meet Analysis Criteria

### Bioinformatics Pipeline for Variant Calling

**FIG 2:**
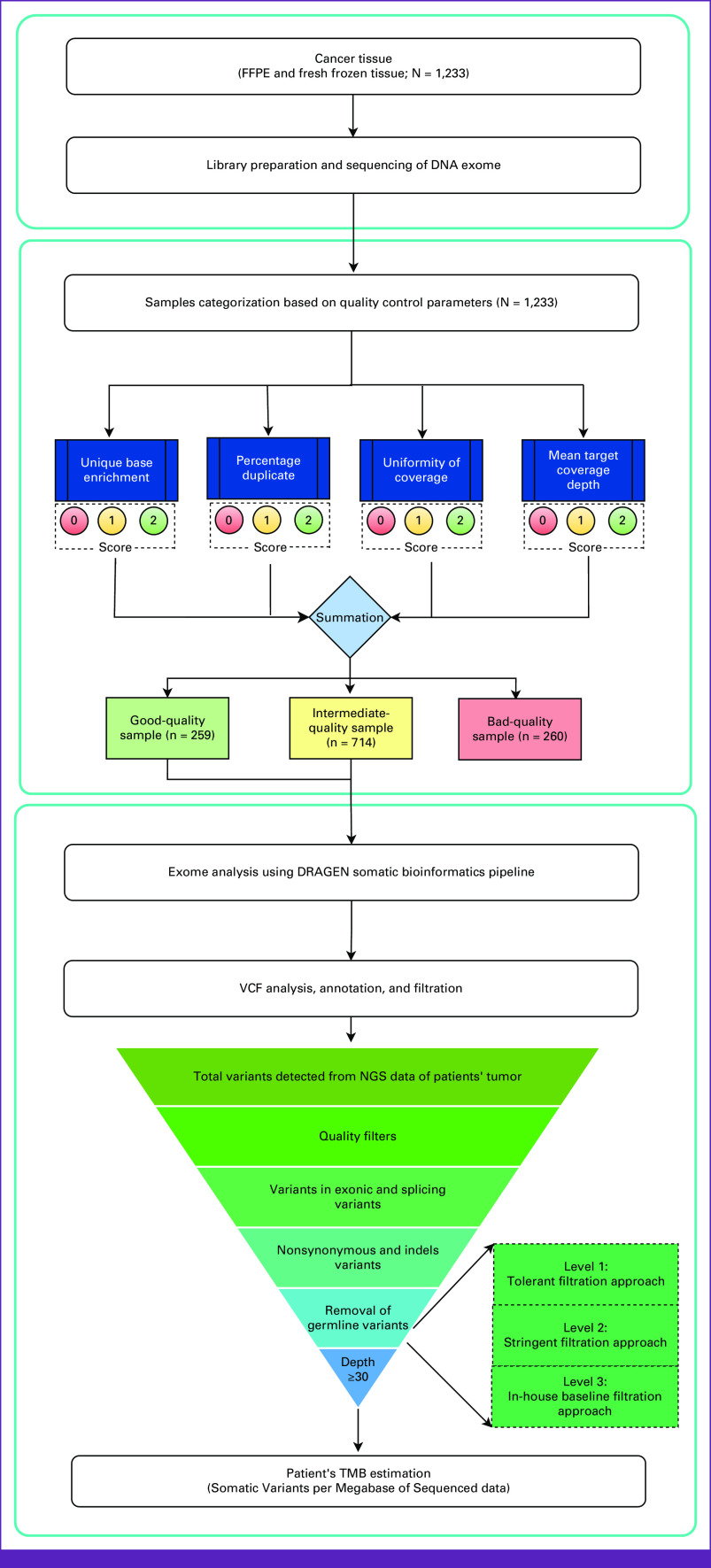
End-to-end workflow from tumor-only samples to estimate the TMB in a clinical setting. This workflow includes library preparation and sequencing, alignment to the human reference genome (GRCh37), quality-based categorization of samples, variant calling for SNV and INDELS, and variant filtration followed by TMB estimation. FFPE, formalin-fixed paraffin-embedded; NGS, next generation sequencing; SNV, single-nucleotide variation; TMB, tumor mutation burden; VCF, variant call format.

The raw sequencing reads were checked for QC using the FastQC tool and trimmed for adapters and a base quality cutoff of Phred score Q30.^15^ High-quality sequencing reads were processed in a comprehensive Illumina DRAGEN Somatic Pipeline (Illumina DRAGEN Bio-IT Platform v3.6) which maps the reads to human reference genome (GRCh37), followed by detection of variants including single-nucleotide variations (SNVs) and small insertions/deletions (INDELs; Fig 2).

### Categorization of the Samples on the Basis of the QC Metrics

**FIG A2:**
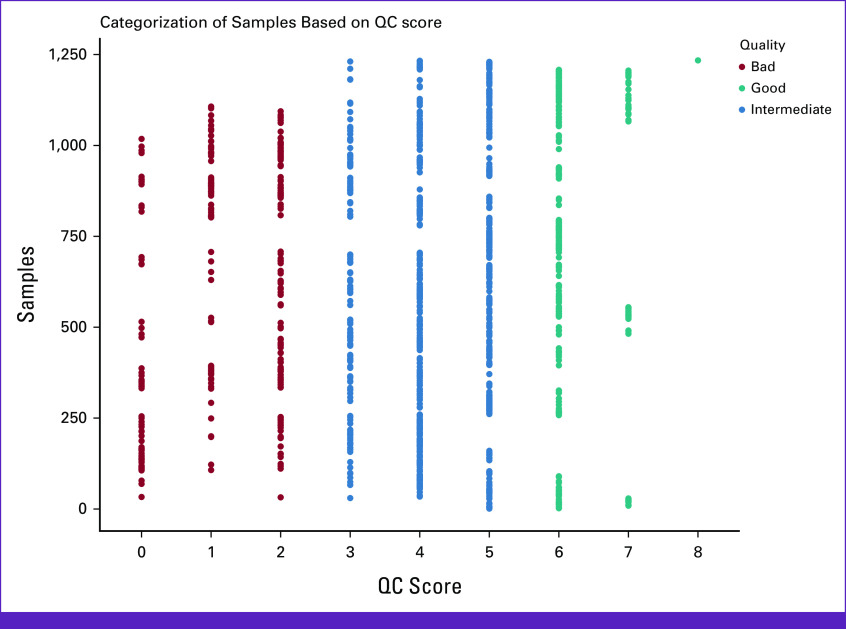
Categorization of samples (N = 1,233) based on summation QC score. Each sample has been scored (0-8 range), where 0 represents that sample has failed in all the QC parameters, and score 8 represents a sample with high-quality data. The samples have been categorized as good (QC score [6-8], represented in green color, n = 259), intermediate (QC score [3-5], represented in blue color, n = 714) and bad quality (QC score [0-2], represented in red color, n = 260). Samples with intermediate and good quality scores were further assessed for TMB estimation. QC, quality control; TMB, tumor mutation burden.

After the initial analysis, four NGS-QC parameters were selected for scoring the samples, which includes the mean target coverage depth, uniformity of coverage, percent duplicate aligned reads, and base enrichment (Table 1).^16^ These parameters were given equal weightage, and each sample was scored as 0, 1, and 2, followed by the calculation of the cumulative scores (Appendix Fig A2). These cumulative scores were further used to categorize each sample as a good (score 6-8), intermediate (score 3-5), and poor (score 0-2) quality sample. After careful consideration, samples with ≥3 QC score were considered as pass and samples with <3 QC score were considered as fail. After applying this filtering approach, the cohort size reduced to n = 973 from N = 1,233 samples. This final subset was used to understand the trends in TMB scores across different cancers in this cohort.

### Variant Annotations and Databases

Variants were annotated using in-house developed pipeline with modules of global and South Asian population databases (gnomAD, 1000G, and ExAC), with an indigenous developed criteria for elimination of germline variants.^17-19^

### Data Acquisition From TCGA

TMB scores of different cancer types were obtained from TCGA. The cancer types included cutaneous melanoma (n = 298), NSCLC (n = 2,206), breast (n = 1,552), sarcoma (n = 741), colorectal (n = 1,353), ovarian (n = 325), pancreatic (n = 849), endometrial (n = 427), CNS (n = 511), prostatic (n = 569), gastric (n = 249), head and neck (n = 174), hepatobiliary (n = 408), renal (n = 201), bladder (n = 232), and esophageal (n = 138).^12^

## RESULTS

### TMB Calculation Workflow Establishment

The TMB calculation workflow was established by using three unique stages, wherein different aspects of the variants that include quality, nature, type, and clinical significance of the variant were considered to rank a variant as a true somatic variant. After this, the total number of true somatic mutations was divided by the size of the exome panel to obtain the TMB score.

### Stage 1—High-Quality Coding Variants Filtration

The detected variants (SNVs/INDELs) were systematically filtered on the basis of variant location and nature of variant type. Only high confidence variants with a minimum quality of 10 (quality score from Illumina DRAGEN Bio-IT Platform v3.6) and a minimum depth of 30× at variant location were considered for the analysis (Fig 2). Synonymous variants were removed, and coding variants were considered for the downstream analysis.

### Stage 2—Germline Filtration

The germline variants were removed using a sequential three-level filtering approach adapted from Parikh et al,^20^ 2020. In level 1 (tolerant approach), the global population frequency and South Asian frequency were used to remove the polymorphic variants (>1% of the population) from the cohort. In level 2 (stringent approach), the value of variant allele frequency was used to remove the germline heterozygous and homozygous variants. Here, variants with allele frequencies ≤ 0.05 and 0.5 ± 0.05 were removed to eliminate the germline variants. In level 3 (baseline approach), variants were filtered on the basis of the in-house baseline (germline samples). The baseline (reference genome pattern) was created by pooling of germline variants derived from WES data from healthy individuals (4baseCare unpublished data; Fig 2).

### Stage 3—Statistical Approach for Tumor-Only Samples

**FIG 3:**
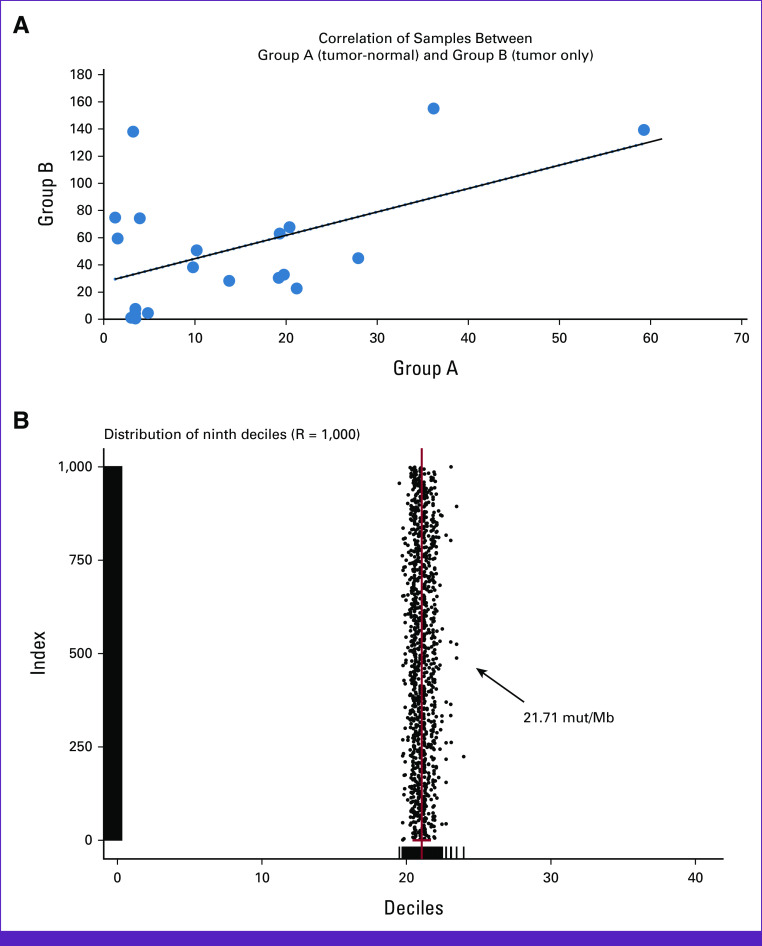
(A) Correlation of TMB scores between group A: tumor normal (n = 20 samples) and group B: tumor only (n = 20 samples) depicts correlation coefficient, *r*^2^ = 0.94. (B) Statistical approach using percentile distributions of TMB scores and bootstrap resampling (machine learning unsupervised approach) depicts clustering of TMB score at the ninth decile with an average TMB score of 21.71 mutation/Mb. TMB, tumor mutation burden.

Variant calling from tumor-only samples may include both rare germline and somatic variants resulting in overestimated TMB scores. To remove the bias and germline variants and further validate the performance of the TMB filtration strategy, we used a training set of 20 matched tumor-normal samples (group A) and 20 tumor-only samples (group B). In group A, germline variants were removed by subtracting the variants from FFPE using matched blood samples to get true somatic variants. However, in group B, germline variants were removed by a three-level filtering strategy (Fig 2). In both groups, all the other variables (confidence value, quality parameters, and depth) were kept constant. The TMB scores of group A (tumor-normal pair) and group B (tumor only) depicted a similar trend. Independent test data of additional 40 samples (group C: 20 tumor-normal and group D: 20 tumor-only) were used to accurately predict the TMB score from tumor-only samples. The Pearson correlation coefficient between tumor-only and tumor-normal samples was determined to be 0.94, which depicts a high correlation between the two groups (Fig 3A). On the basis of this validation analysis, we were able to establish confidence and robustness in our TMB workflow that could be implemented in the remaining clinical samples of the cohort.

The count for true somatic variants was derived using the group B algorithm (tumor-only workflow), and it was divided by the target size to estimate the TMB score for a given tumor sample.

### Trend Analysis of TMB Scores Using Bootstrap Resampling Approach From Indian Cohort

In the context of cancer genome landscape, somatic mutations are the primary variables that contribute to interpatient variability and hence the TMB scores.^21^ We adopted a statistical model using the percentile distributions of TMB scores and a bootstrap resampling approach using the base package in R (The R Foundation for Statistical Computing, Vienna, Austria).^22^ In this unsupervised approach, 1,000 iterations of a phantom data set (randomly resampled cohorts) were generated from the primary cohort (n = 973; QC score ≥3). This phantom data set was used to calculate the average TMB score at ninth decile (same as 90th percentile) which was observed to be 21.71 mutation/megabase (mut/Mb; Fig 3B).

### Distribution of TMB Across Cancer Types in Indian Cohort

**FIG 4:**
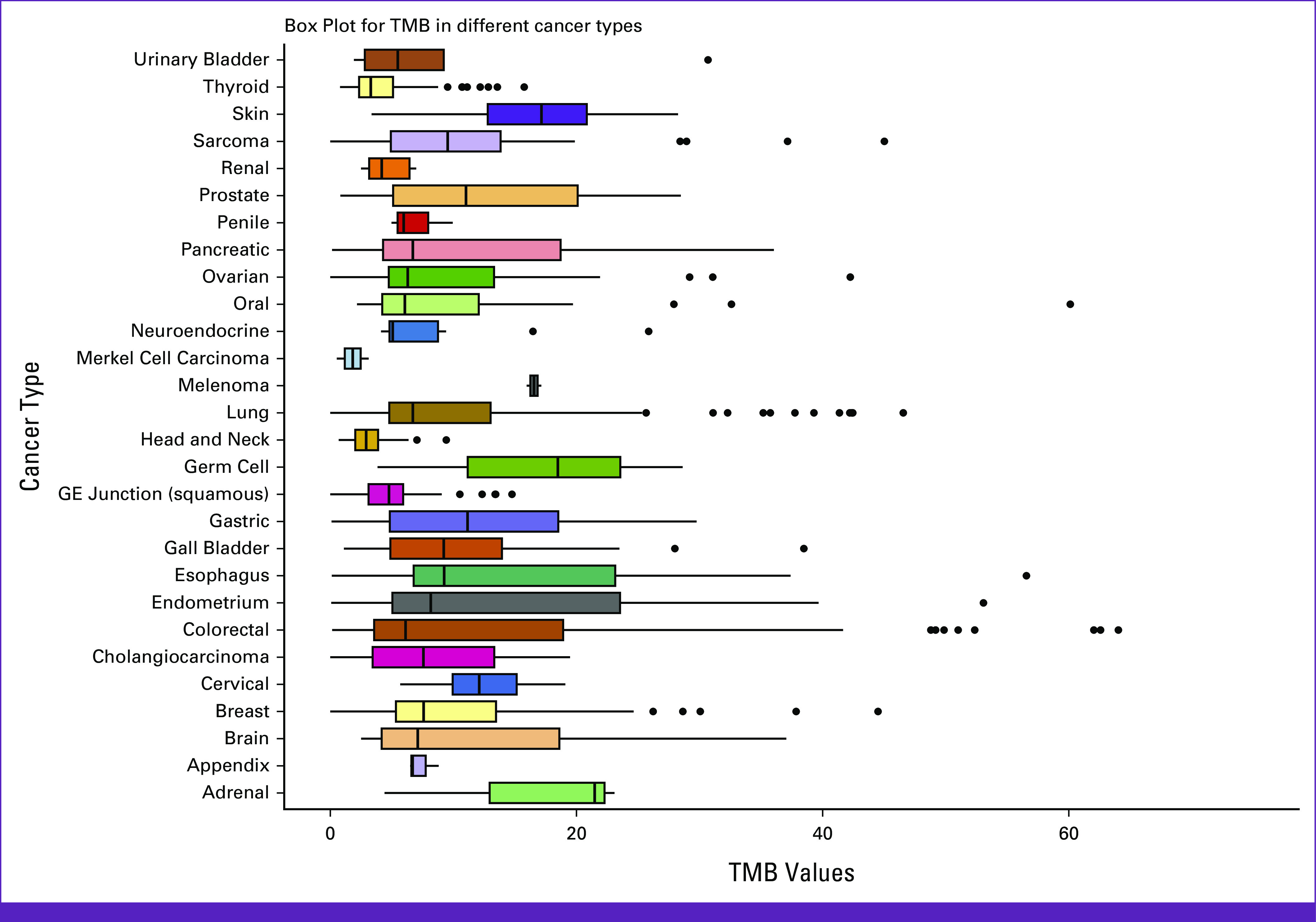
TMB varies among cancer types from Indian cohort: Distribution of TMB scores of n = 973 (QC score ≥3, QC passed samples) patients across 28 (excluding mixed and rare cancer types with less representation) different tumors from Indian cohort using box plot. The bottom of the box represents the 25th percentile, and the top of the box represents the 75th percentile. GE, gastroesophogeal; QC, quality control; TMB, tumor mutation burden.

In this study, we have noticed a broad distribution for TMB in the range of 0-161.25 mut/Mb, which varies significantly across different cancer types within the cohort. TMB scores in the brain (range, 2.51-161.26 mut/Mb; median, 9.04 mut/Mb), colorectal (range, 0.15-64.02 mut/Mb; median, 6.11 mut/Mb), oral (range, 2.17-60.12 mut/Mb; median, 6.06 mut/Mb), esophagus (range, 0.12-56.55 mut/Mb; median, 9.24 mut/Mb), endometrial (range, 0.08-53.06 mut/Mb; median, 8.16 mut/Mb), breast (range, 0-44.5 mut/Mb; median, 7.58 mut/Mb), and lung (range, 0-46.55 mut/Mb; median, 6.71 mut/Mb) depict a broad range TMB. In contrast, cancers such as renal (range, 2.49-6.97 mut/Mb; median, 4.18 mut/Mb) and head and neck (range, 0.68-9.42 mut/Mb; median, 2.91 mut/Mb) have shown restricted distribution (Fig 4).

**FIG 5:**
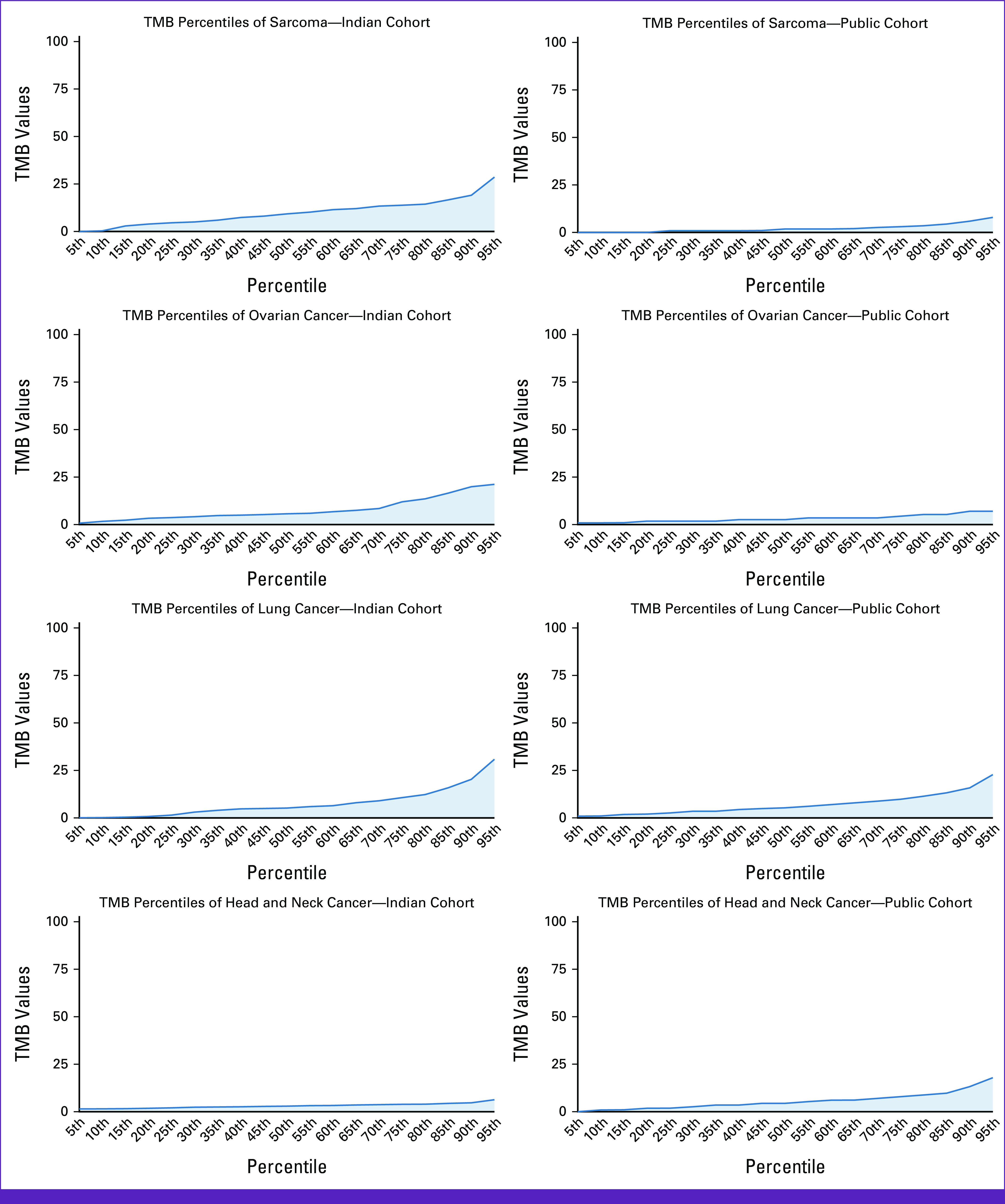

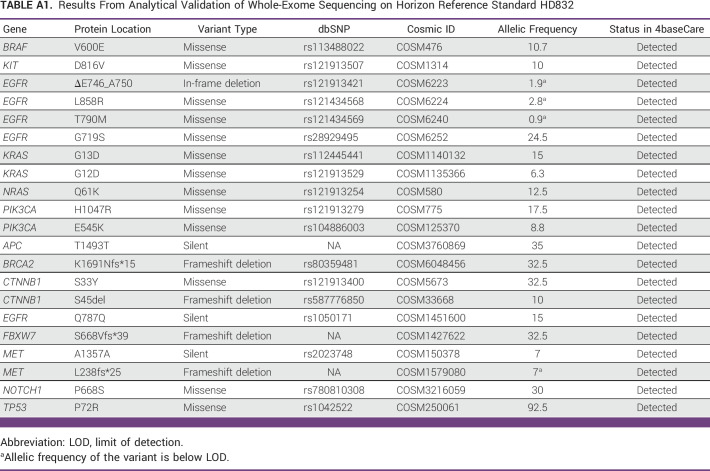
Distribution of TMB score percentile: Image showing the distribution of TMB scores across six different cancer type (n ≥ 30 for each cancer type) in Indian and TCGA cohort. TCGA, The Cancer Genome Atlas; TMB, tumor mutation burden.

**FIG A3:**
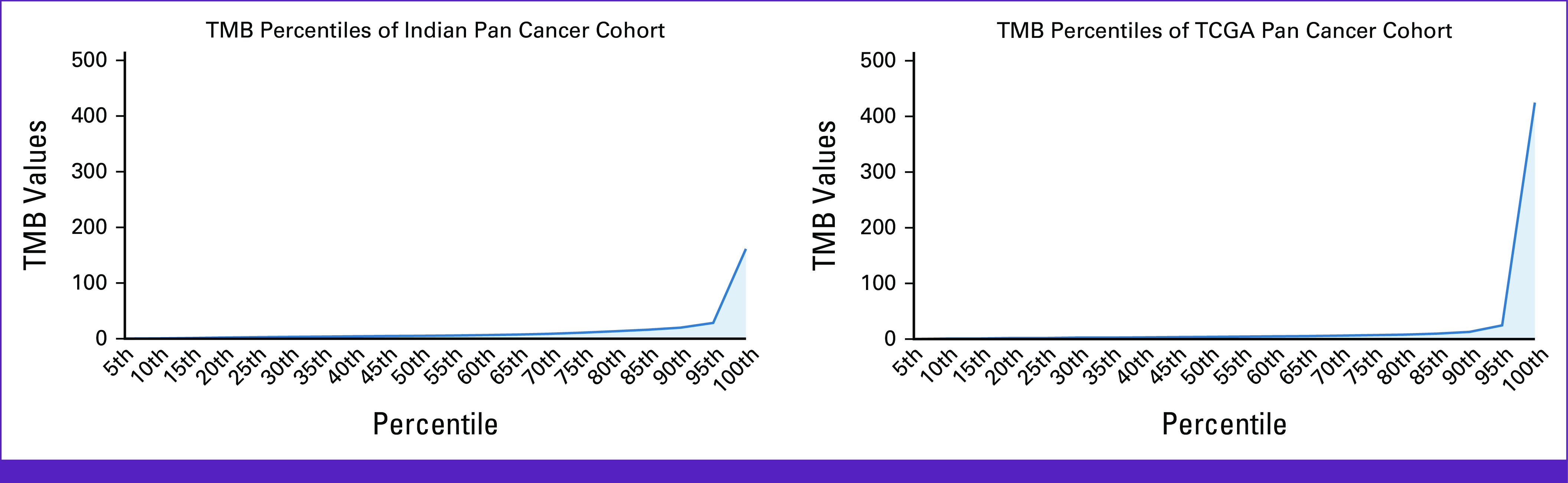
TMB score percentile distribution across (A) Indian Pan-cancer and (B) TCGA Pan-Cancer. TCGA, The Cancer Genome Atlas; TMB, tumor mutation burden.

The methodology of percentile distribution helps to understand the biology behind the stratification of the cases on the basis of the TMB scores. Using this percentile approach, we have studied the distribution of TMB scores for pan-cancer and in individual cancers (n ≥ 30): sarcoma, ovary, lung, head and neck, breast, and colorectal from Indian patients. We have observed a biphasic trend in the pan-cancer distribution of TMB scores (Appendix Fig A3). In addition, the pattern of the TMB distribution varies among different cancer types between the Indian and TCGA cohort (Fig 5).

### Comparison of TMB Score Distribution Between Indian and TCGA Cohort Across Six Cancer Types

The TMB range varies in the Indian cohort (0-161.25 mut/Mb range) as compared with the TCGA cohort (0–424.8 mut/Mb). We overlapped the percentile distribution along with the median of TMB scores (calculated using Kruskal-Wallis test) in these two cohorts (n ≥ 30 for each cancer type). The most critical finding from this study demonstrates a significantly different TMB score distribution between the two population cohorts; particularly, it was evident in four different cancer types: sarcoma, ovarian, head and neck, and breast. Nevertheless, in lung and colorectal cancer, surprisingly, we observed a similar score and trend distribution. (Detail of the analysis have been summarized in the Data Supplement.) Our observation might provide some clue to explain the differences in underlying biology and hence the spectrum of mutations and their evolution between the two populations (Fig 5).

## DISCUSSION

Clinical outcomes in patients with cancer vary across geographies, which is a well-known fact from several studies.^23-25^ TMB is one such predictive immunotherapy biomarker that has gained importance in the past 5 years after the FDA approval of pembrolizumab.^26^ However, the pan-cancer TMB score threshold as a predictive biomarker has remained a limitation in patient stratification on the basis of the outcome data from recent clinical studies.^27^ A review from Japanese Society of Medical Oncology/Japan Society of Clinical Oncology/Japanese Society of Pediatric Hematology/Oncology suggested that optimal TMB cutoff differ according to the cancer type.^28^ Hence, it is important to define TMB subgroups using an appropriate threshold for individual tumors, rather than a fixed number threshold across all cancers.

There are growing evidences to demonstrate the importance of incorporating ethnicity and geographic variability as confounding factors while estimating the TMB. As mentioned earlier, a study conducted by Nassar et al^10^ shed light on recalibration of existing TMB with ancestry-driven TMB for better outcome. Another study by Starks et al used 1,047 patients from 10 ethnicities where she employed a mitigation strategy to use ancestry-driven gene panels, population-specific variant filtration, removal of germline variant, and heuristic population-neutral target selection. Interpopulation comparison of TMB before and after the approach had a significant difference in the mutation signature of the patients, which indicates the need of unbiased bioinformatics approach for each population and ancestry.^29^ In our study, we have taken steps to remove population-specific polymorphism and germline variants by deploying the three-step filtration strategy. Therefore, assuming a universal criterion and a cutoff for all ethnicities and geographical area may have adverse effects on the patient with cancer.

In this study, we have profiled Indian patients with cancer using WES to estimate the TMB scores and observed variation in the distribution of TMB scores in different cancer types. As an example, head and neck and renal cancer fall into the category of a narrow range of TMB scores (≤10 mut/Mb), whereas breast, lung, colon, pancreatic, and others fall into the category of a broad range of TMB scores (0-161.26 mut/Mb).

Comparative analysis of TMB scores between Indian and TCGA cohorts depicted higher TMB scores in few cancers of Indian patients as compared with TCGA counterparts (Fig 5). The Kruskal-Wallis test was used to identify the difference in the median for six different cancer types between the two cohorts. It was observed that there was a significant difference in median, TMB score distribution, and the proportion of patients, who may respond to ICI across four cancer types (sarcoma, ovary, head and neck, and breast). In contrast, similar scores were observed across the 75-95th percentile in lung cancer indicating that the efficacy of ICI therapy in patients with lung cancer may not vary significantly between the Indian and TCGA cohorts. As we carefully examined TMB score distribution at the 95th percentile, we observed a trend of higher cut-off for the selection of patients in the Indian cohort as compared to TGCA for the following cancer types: sarcoma, ovary, and breast. However, we observed similar trends in lung and colorectal, while in head and neck we observed lower score cut-off in Indian data as compared to TCGA (Fig 5). In other words, the thresholds that determine the responders to ICI on the basis of the TMB value may be different between the Indian and the TCGA cohort. In summary, our findings reiterate the need of establishing population-specific and cancer-specific TMB thresholds for the stratification of patients with cancer for ICI. In addition, this study indicates that TMB scores can be calculated accurately on the basis of the tumor-only NGS data to reduce the cost burden for these patients with cancer. It may also provide leads to act on unique pathways that drive cancers on the basis of their genomic signatures and mutational patterns.

As per our knowledge, this publication is the first report from India to understand the TMB score distribution across multiple cancers in a large cohort of Indian patients with cancer (N = 1,233). Considering the cancer burden and heterogeneity in India, our cohort of patients is still a biased population because of a random collection of samples from different parts of India for various cancer types. Several technical factors such as tissue processing, representative tumor material, choice of NGS panel, bioinformatics pipeline, stage of the disease, and variant filtration strategy could affect the TMB score calculation. Future studies on a much larger cohort of patients with cancer with adequate representation for all the rare cancers, such as sarcomas, gliomas, and others, may throw some light on TMB and its role in predicting ICI response in these cancers.

## Figures and Tables

**TABLE A1:**
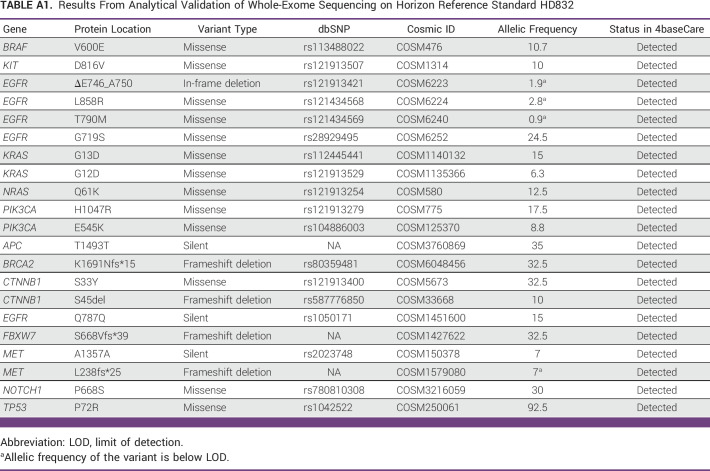
Results From Analytical Validation of Whole-Exome Sequencing on Horizon Reference Standard HD832

**TABLE A2:**
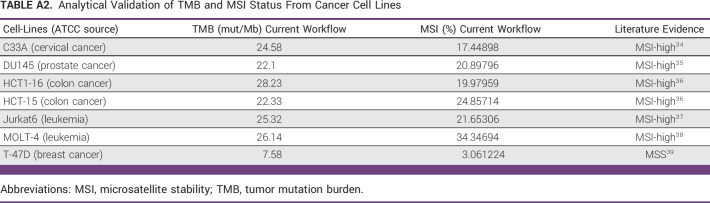
Analytical Validation of TMB and MSI Status From Cancer Cell Lines

## APPENDIX 1. SUMMARY

Tumor mutation burden (TMB) is a mathematical calculation that involves the total number of somatic mutations in a given patient's tumor divided by the size (in Mb) of the gene panel used for profiling. Our study design has adequately demonstrated the TMB analytical workflow on the basis of the TMUGS (Tumor Marker Utility Grading System) recommendation and REMARK (REporting recommendations for tumor MARKer prognostic studies) criteria from ASCO.^30,31^

### Introduction

Whole-exome sequencing (WES) is considered as the gold standard for evaluating TMB as it provides comprehensive coverage of the coding region of the human genome. It is already established by multiple studies that the calculation of TMB score is not only influenced by the size of the sequencing panel and the choice of genes but also by the bioinformatics workflow used in different laboratories. Variations in TMB calculations across laboratories can also be due to differences in genomic diversity in the population, as well as variability in cancer risk predisposition and its etiology.

In the proposed approach, the impact of population-specific diversity in polymorphisms and rare variants is incorporated into the variant filtration strategy. This is believed to provide a more accurate TMB score and better stratification of patients with cancer for the selection of immune checkpoint inhibitors, reducing toxicity and lowering the health care cost burden for the patient.

### Study Design

This research was a retrospective observational study of patients with cancer WES data collected between 2020 and 2022. TMB calculation is based on archived tumor specimens: FFPE blocks. According to recommendations from REMARK, the adequacy of tumor content was maintained uniformly by enforcing a strict cutoff of >10% tumor load, with a minimum of 150 viable tumor cells per high-power field (HPF) and a minimum of two HPFs in the hematoxylin and eosin examination of the specimen. Only specimens that met these criteria were considered for further processing.

### Analytical validation

The basic framework of variant calling followed by deriving true somatic variant from WES data from FFPE was established using DRAGEN-Bio IT platform and indigenous developed filtration strategy (Fig 2 of original article). The analytical validation of the workflow was performed in two stages:

1. Robustness of variant calling pipeline using Horizon reference standard HD832

Appendix Table A1 displays the outcomes from the Horizon Reference Standard, which demonstrates the capability of the WES pipeline to identify variants with a limit of detection (LOD) of 5% for SNVs and 10% for INDELs.

2. Robustness of estimation of genomic signature (TMB and MSI) using seven different sources of DNA across multiple cancers

Appendix Table A2 summarizes the MSI and TMB calculation using WES data for six cancer cell lines (C33A, DU145, HCT-15, HCT-116, Jurkat6, and MOLT-4) with documented MSI-high status. The MSI scores are >22% and the TMB scores are above 18 mutations/Mb, supporting previous clinical studies. The T-47D breast cancer cell line was earlier established as MSS cell line and same was reproduced as MSS (7.58%) and TMB-low (3 mutations/Mb). The MSI calculation workflow is based on algorithm MSI-sensor2.^32^ Extrapolating the vast literature evidence that shows a strong correlation between MSI-high and TMB-high,^33^ we tried to derive similar conclusion in our analytical validation of MSI and TMB. Out internal threshold from MSI score being high is >15% and current research on pan-cancer TMB distribution showed 21.71 mut/Mb at ninth decile.

In summary, the performance metrics of analytical validation of TMB workflow that included true somatic variant calling demonstrates 98% specificity, 100% sensitivity, and 100% reproducibility at a LOD of 5% for SNVs and 10% for INDELs.
